# Supplementary material for: Evidence of potential overdiagnosis and overtreatment of attention deficit hyperactivity disorder (ADHD) in children and adolescents: protocol for a scoping review
Source: BMJ Open. 2019 Nov 7;9(11):e032327. doi: 10.1136/bmjopen-2019-032327 (PMC6858259; doi:10.1136/bmjopen-2019-032327)
Supplement: Supplementary data [file bmjopen-2019-032327supp002.pdf]

SUPPLEMENT II

MEDLINE SEARCH STRATEGY

**0. Is ADHD overdiagnosed?**	**1. Is there potential for increased diagnosis?**	**2. Is diagnosis actually increased?**	**3. Are additional cases subclinical or low risk?**	**4. Are additional cases treated?**	**5.a) Might harms outweigh benefits for treatment?**	**5.b) Might harms outweigh benefits for diagnosis?**
((exp Attention Deficit Disorder with Hyperactivity/ OR adhd.ti. OR hyperkinesis.ti. OR exp Hyperkinesis/ OR Attention Deficit Hyperactivity Disorder.ti. OR Attention Deficit Disorder with Hyperactivity.ti. OR Hyperkinetic Disorder.ti.)						
AND						
(Child*.tw. OR Child/ OR Adolescen*.tw. OR exp Adolescent/ OR exp Infant/ OR Infan*.tw. OR Minors/ OR p?ediatric*.tw. OR Pediatrics/ OR primary school*.tw. OR school*.tw. OR kindergarten.tw. OR pre-school.tw. OR Pre School.tw. OR elementary school.tw. OR student*.tw. OR secondary school.tw. OR Schools/ OR high school*.tw. OR Child Psychiatry/ OR Adolescent Psychiatry/)						
AND						
(overdiagnos*.mp. OR over diagnos*.mp. OR overtest*.mp. OR over test*.mp. OR exp Medical Overuse/ OR overuse*.mp. OR over use*.mp. OR overdetect*.mp. OR over detect*.mp. OR insignificant disease.mp. OR overtreat*.mp. OR over treat*.mp. OR inconsequential disease.mp. OR overmedical*.mp. OR unnecessary procedure*.mp. OR exp Unnecessary Procedures/ OR pseudodisease.mp. OR pseudo disease.mp. OR "too much medicine".mp. OR nondisease.mp. OR non	((continuum OR continual* OR continuous* OR dimension* OR categoric* OR spectrum OR subthreshold OR threshold OR full syndrome OR dichotomous OR linear association OR distribution of symptom* OR full symptom*).tw))	(prevalence/ OR prevalen*.ti. OR incidence/ OR inciden*.ti. OR frequency.ti. OR rate.ti. OR definition*.ti. OR diagnos*.ti. OR Diagnosis/ OR phenotype.ti)	(severity.tw. or impair*.tw. or mild.tw. or moderate.tw. or severe.tw. or extreme.tw. or subclinical.tw. or subthreshold.tw)	(treatment*.ti. or exp Therapeutics/ or medication*.ti. or pharma*.ti. or Pharmaceutical Preparations/ or Ritalin.ti. or exp Methylphenidate/ or Central Nervous System Stimulants/ or stimulant*.ti. or drug*.ti. or therapeutics/ or drug therapy/ or therapeutic*.ti. or Methylphenidate.ti. or psychostimulant*.ti. or Dexmethylphenidate.ti. or Dexmethylphenidate Hydrochloride/ or Atomoxetine*.ti. or Atomoxetine Hydrochloride/ or nonpsychostimulant.ti. or exp Amphetamines/ or amphetamine*.ti. or adderrall.ti or antipsychotic*.ti. or Antipsychotic Agents/)		(label*.mp.))
	NOT	AND	AND	AND	AND	NOT
	(Autism.ti. OR Autistic.ti. OR exp Autistic Disorder/ OR exp Autism spectrum Disorder/)	(trend*.tw. OR field trial*.tw. OR follow-up studies/ OR follow-up.tw. OR chang*.tw. OR	(prevalence/ OR prevalen*.ti. OR incidence/ OR inciden*.ti. OR frequency.ti. OR	(trend*.ti. OR change*.ti. OR variation*.ti. OR vary*.ti. OR increase*.ti. OR	(Treatment Outcome/ or outcome*.tw. OR consequence*.tw. OR impact*.tw.) AND	(off-label.tw. OR Open-Label.tw.)

disease.mp. OR "false positive*".mp. OR overdefinition*.mp. OR over definition*.mp. OR misdiagnos*.mp. or Diagnostic Errors OR variation of care.mp. OR medicali*.mp. OR Medicalization/))		variation*.tw. OR vary*.tw. OR increas*.tw. OR decreas*.tw. OR pattern*.tw. OR expan*.tw.))	rate.ti. OR trend*.ti. OR change*.ti. OR variation*.ti. OR vary*.ti. OR increase*.ti. OR decrease*.ti. OR pattern*.ti. Or expand*.ti.))	decrease*.ti. OR pattern*.ti. Or expand*.ti. OR overprescri*.ti. OR prescri*.ti. OR underprescri*.ti.))	(Patient Harm/ or harm*.tw. OR Cost-Benefit Analysis/ OR benefit*.tw. OR cost*.tw. OR Risk/ or risk*.tw. or improv*.tw. or positive.tw. or negative.tw. OR worse.tw. or better.tw. OR adverse.tw. OR effect*.tw.)	
					AND	
					(((meta analysis or "systematic review").pt.) OR (Cohort.tw. OR longitud*.tw. OR observation*.tw. OR follow-up.tw. OR registries/ OR longitudinal studies/)))	
NOT						
((autobiography OR bibliography OR biography OR case reports OR comment OR congress OR consensus development conference, nih OR dataset OR dictionary OR directory OR editorial OR expression of concern OR festschrift OR government document OR guideline OR interactive tutorial OR lecture OR legal case OR legislation OR letter OR news OR newspaper article OR patient education handout OR personal narrative OR portrait OR scientific integrity review OR technical report OR video-audio media).mp. OR webcasts.pt.)						
AND						
limit search to (english language and yr="1979 -Current")						
