# Supplementary material for: Evidence of potential overdiagnosis and overtreatment of attention deficit hyperactivity disorder (ADHD) in children and adolescents: protocol for a scoping review
Source: BMJ Open. 2019 Nov 7;9(11):e032327. doi: 10.1136/bmjopen-2019-032327 (PMC6858259; doi:10.1136/bmjopen-2019-032327)
Supplement: Supplementary data [file bmjopen-2019-032327supp003.pdf]

SUPPLEMENT III

DRAFT DATA COLLECTION ITEMS

Source

- Author
- Title
- Year
- Citation

Eligibility

- Reason for exclusion
- Reason for inclusion
- Answers which question (1-5)

Methods

- Study design
- Study Question/Aim

Population Characteristics

- Number
- Setting
- Age
- Sex
- Country
- Co-morbidity
- Socio-demographics/ethnicity/other

Intervention/Exposure

- Specific Intervention/Exposure
- Comparator
- Number of groups

Outcomes

- Outcomes
- Definition of outcomes/diagnostic criteria used
- Scales/threshold

Results

- Estimate of effect, CIs, p-values

Miscellaneous

- Funding
- Key conclusions
- Comments
- Relevant References
