# Supplementary material for: Evidence of potential overdiagnosis and overtreatment of attention deficit hyperactivity disorder (ADHD) in children and adolescents: protocol for a scoping review
Source: BMJ Open. 2019 Nov 7;9(11):e032327. doi: 10.1136/bmjopen-2019-032327 (PMC6858259; doi:10.1136/bmjopen-2019-032327)
Supplement: Author's manuscript [file bmjopen-2019-032327.draft_revisions.pdf]

BMJ Open

BMJ Open is committed to open peer review. As part of this commitment we make the peer review history of every article we publish publicly available.

When an article is published we post the peer reviewers' comments and the authors' responses online. We also post the versions of the paper that were used during peer review. These are the versions that the peer review comments apply to.

The versions of the paper that follow are the versions that were submitted during the peer review process. They are not the versions of record or the final published versions. They should not be cited or distributed as the published version of this manuscript.

BMJ Open is an open access journal and the full, final, typeset and author-corrected version of record of the manuscript is available on our site with no access controls, subscription charges or pay-per-view fees (http://bmjopen.bmj.com).

If you have any questions on BMJ Open's open peer review process please email info.bmjopen@bmj.com

BMJ Open

Overdiagnosis and overtreatment of Attention Deficit Hyperactivity Disorder (ADHD) in children and adolescents: protocol for a scoping review of the evidence

Journal:	*BMJ Open*
Manuscript ID	bmjopen-2019-032327
Article Type:	Protocol
Date Submitted by the Author:	13-Jun-2019

Complete List of Authors:	Kazda, Luise; The University of Sydney, Sydney School of Public Health<br>Bell, Katy; The University of Sydney Faculty of Medicine and Health, School of Public Health; The University of Sydney<br>Thomas, Rae; Bond University, Faculty of Health Sciences and Medicine<br>McGeechan, Kevin; The University of Sydney, School of Public Health;<br>Barratt, Alexandra; University of Sydney, School of Public Health

Keywords:	EPIDEMIOLOGY, MENTAL HEALTH, PAEDIATRICS, Child & adolescent psychiatry < PSYCHIATRY, PUBLIC HEALTH

SCHOLARONE™
Manuscripts

Overdiagnosis and overtreatment of Attention Deficit Hyperactivity Disorder (ADHD) in children and adolescents: protocol for a scoping review of the evidence

Luise Kazda[1*], Katy Bell[1], Rae Thomas[2], Kevin McGeechan[1], Alexandra Barratt[1]

[1]Sydney School of Public Health, Faculty of Medicine and Health, The University of Sydney, NSW, Australia

[2]Institute for Evidence-Based Healthcare, Bond University, Gold Coast, Qld, Australia

*Luise Kazda

Room 124, Edward Ford Building (A27), The University of Sydney NSW 2006, Australia

luise.kazda@sydney.edu.au

+61 2 90365360

Word count: 2,231

Key Words: Attention Deficit Hyperactivity Disorder (ADHD), overdiagnosis, overtreatment, children, adolescents

For peer review only - http://bmjopen.bmj.com/site/about/guidelines.xhtml

Title

Overdiagnosis and overtreatment of Attention Deficit Hyperactivity Disorder (ADHD) in children and adolescents: protocol for a scoping review of the evidence

Abstract

Introduction

Worldwide, ADHD diagnosis rates in children and adolescents have been increasing consistently over the past decades, fuelling a debate about the underlying reasons for this trend. While many hypothesise that a substantial number of these additional cases are overdiagnosed, to date there has been no comprehensive evaluation of evidence for or against this hypothesis. Thus, with this scoping review we aim to synthesise published evidence on the topic, in order to investigate whether existing literature is consistent with the occurrence of overdiagnosis and/or overtreatment of ADHD in children and adolescents.

Methods and Analysis

The proposed scoping review will be conducted in the context of a framework of five questions, developed specifically to identify areas in medicine with the potential for overdiagnosis and overtreatment. The review will adhere to the Joanna Briggs Methodology for Scoping Reviews. We will search MEDLINE, Embase, PsychINFO, and the Cochrane Library electronic databases for primary studies published in English from 1979 onwards. We will also conduct forward and backward citation searches of included articles. Data from studies that meet our pre-defined exclusion and inclusion criteria will be charted into a standardised extraction template with results mapped to our pre-determined five questions framework in form of a table and summarised in narrative form.

Ethics and Dissemination

The proposed study is a scoping review of the existing literature and, as such, does not require ethics approval. We intend to disseminate the results from the scoping review through publication in a peer-reviewed journal and through conference presentations. Further, we will use the findings from our scoping review to inform future research to fill key evidence gaps identified by this review.

For peer review only - http://bmjopen.bmj.com/site/about/guidelines.xhtml

For peer review only

For peer review only - http://bmjopen.bmj.com/site/about/guidelines.xhtml

STRENGTHS AND LIMITATIONS OF THIS STUDY

- The planned study will be the first comprehensive synthesis of published evidence on the potential for overdiagnosis and overtreatment in ADHD in children and adolescents.
- We will use a broad and systematic search strategy to retrieve potentially relevant studies, even where these do not explicitly use the terms "overdiagnosis" or "overtreatment" (or variations of these).
- Our review will identify gaps in the current evidence and recommendations for further necessary research in the field.
- By applying and testing the five indicators in a new condition, we will provide further validation of the usefulness of these indicators of potential overdiagnosis.
- As is typical with a scoping review, we will not undertake formal risk of bias assessment of included studies; however, an appraisal of study quality will be integrated into the narrative synthesis of the evidence.

For peer review only

INTRODUCTION

Review Questions

Our primary study question for this scoping review is: "Does the published literature indicate a potential for overdiagnosis and/or overtreatment of Attention Deficit Hyperactivity Disorder (ADHD) in children and adolescents?" Besides this question, we will consider two further, secondary questions in our review: "When mapped against an existing framework for identifying potential overdiagnosis[1], are there any gaps in the current evidence?" and "How does the framework[1] perform in terms of applicability and usability for identifying potential overdiagnosis of a mental health condition?"

Background

With steadily increasing prevalence rates of ADHD throughout the developed world[2-6], there is growing debate about whether this trend is due to an actual increase in prevalence, better detection and diagnosis, misdiagnosis, or overdiagnosis[7-10]. Whilst the evidence for overdiagnosis in many cancers is increasingly recognised[11 12], the evidence for overdiagnosis of ADHD, a non-cancer condition where overdiagnosis is widely thought to occur, has not yet been comprehensively evaluated[13]. Quantifying the potential for overdiagnosis is only just emerging as a field of interest in both paediatric and mental health research[7 14].

Overdiagnosis of ADHD appears to be a result of three main drivers. Firstly, it can arise due to the problem of overdefinition[15], that is, lowering the threshold for a disease, by expanding the disease definition to include people with ambiguous or very mild symptoms without evidence that doing so improves patients' health overall and in the longer term[16 17]. Secondly, overdiagnosis may also be caused by overdetection[4 18] (e.g., screening children at young ages for behaviour problems) and, thirdly, by the medicalisation of some behaviour patterns (e.g. those typical of relatively younger school children)[19]. Other factors may also have played a part. These include formal changes to the diagnostic threshold (DSM-V vs DSM-IV), pharmaceutical industry influence[13] and health and social service drivers (e.g. access to resources linked to a diagnosis)[10].

Although the prevalence rates of ADHD seem to have risen substantially in the past few years, most cases continue to be reported as mild to moderate forms (87% in 2007, 84% in 2011 and 86% in 2016 of all reported cases in a large US based survey)[2 20] and it has been argued that

For peer review only - http://bmjopen.bmj.com/site/about/guidelines.xhtml

many of those cases may represent overdiagnosis. Should this be the case, these children may not experience a net benefit from an ADHD diagnosis and subsequent treatments, but may be harmed[13].

While correctly diagnosing and treating ADHD has many potential benefits[21] the harms from overdiagnosing and overtreating ADHD are significant and costly on multiple levels. Not only may the individual child experience negative physical and psychosocial effects, their families may also experience psychosocial and financial burdens. Overdiagnosis and overtreatment also results in financial and opportunity costs to the health system, and to society at large[7].

Rationale

In summary, while there are increasing concerns about the potential for overdiagnosis of ADHD in children and adolescents, there is scant evidence quantifying the problem. Consequently, a systematic review of the literature to quantify ADHD overdiagnosis is not possible due to the current lack of synthesisable evidence in this field. The rationale for this broader scoping review then, is to use a recently developed framework of questions[1] to systematically determine if the existing literature indicates a potential for overdiagnosis and overtreatment in ADHD. We hypothesize that ADHD fulfils these pre-determined criteria for potential overdiagnosis. A secondary aim is to further examine and highlight any gaps in the current evidence that may prevent us from determining whether or not ADHD is overdiagnosed and overtreated. This aim will be especially helpful in guiding subsequent research in this area. A further secondary aim of the study is to rigorously test the existing 5-questions-framework[1] for its applicability and usability in another area, namely paediatric mental health.

Preliminary searches of the literature were conducted in March and April 2019 to determine if any previous scoping or systematic reviews had been conducted or protocols submitted which aimed to summarise existing evidence on overdiagnosis and overtreatment in ADHD. Databases searched were the Cochrane Database of Systematic Reviews, PROSPERO, MEDLINE, Scopus and the JBI Database of Systematic Reviews and Implementation Reports. Two scoping reviews on overdiagnosis in health care were found but neither review was focused on overdiagnosis and overtreatment of ADHD. One review covered the drivers of overdiagnosis and potential solutions[22], while the other addressed overdiagnosis across

For peer review only - http://bmjopen.bmj.com/site/about/guidelines.xhtml

different medical disciplines[22 23]). Whilst a number of systematic reviews have been conducted on ADHD, these have been restricted to prevalence[24-26] or treatment options[27 28]. We also identified one systematic review on over- or under-prescribing of ADHD medication that is currently underway with a published protocol[29]. Literary reviews and commentaries on overdiagnosis in mental health, including ADHD have been published[13 21 30]. However, we found no reviews that have been conducted or are in preparation, that systematically gather and analyse all available evidence to allow a comprehensive assessment on the potential for overdiagnosis of ADHD.

METHODS AND ANALYSIS

The proposed review will follow the Joanna Briggs Methodology for Scoping Reviews[31] which is based on and extends the work of Arksey and O'Malley[32] as well as that of Levac and colleagues[33]. The scoping review will also adhere to the Preferred Reporting Items for Systematic Reviews and Meta-Analyses Extension for Scoping Reviews (PRISMA-ScR)[34] (Supplement I). This approach was chosen to document the review process as clearly and rigorously as possible.

Inclusion Criteria

Participants

The scoping review will include studies whose main focus is on children and adolescents under the age of 18, who have either been clinically diagnosed or identified by parent – teacher – or self-report as having behavioural symptoms of ADHD. Articles that have a clear emphasis on adult ADHD will be excluded unless they are longitudinal follow-up studies where participants were determined to have ADHD in childhood and were then followed through into adulthood. Moreover, studies with other health issues or disabilities as the primary focus will also be excluded. However, studies considering other mental health comorbidities as a secondary diagnosis will be eligible for inclusion.

Concept

The two core concepts to be examined by this scoping review are "overdiagnosis of ADHD" and "overtreatment of ADHD". Debate over the definition of overdiagnosis is ongoing[1 16 17 35 36], especially in non-cancer contexts. We define overdiagnosis as occurring where a person is correctly diagnosed (according to contemporary professional standards) with a condition but

For peer review only - http://bmjopen.bmj.com/site/about/guidelines.xhtml

For peer review only

the net effect of the diagnosis for the individual concerned is unfavourable (i.e. when consideration is given to the balance of potential harms and benefits)[1 37]. The resulting overtreatment can then be defined as receiving treatment following an overdiagnosis (or among overdiagnosed individuals)[16 38]. It is important to note that overtreatment can occur as a result of overdiagnosis but also without it, due to other drivers[16]. In this scoping review we will only consider pharmaceutical treatment options for ADHD in terms of overtreatment.

Context

The study will be conducted in the context of a previously published and tested framework of five questions that identify characteristics consistent with the occurrence of overdiagnosis and overtreatment[1]. It is not limited to any geographic areas or settings. The five questions were developed by experts in the field of overdiagnosis as a guide to identifying areas in medicine where overdiagnosis and -treatment may be occurring. Additionally, an overarching primer question will be included to avoid missing evidence. Hence, the questions by which the search will be guided and to which the evidence will be mapped are:

0. Is ADHD overdiagnosed in children and adolescents?
1. Is there potential for increased diagnosis?
2. Is diagnosis actually increased?
3. Are additional cases subclinical or low risk?
4. Are additional cases treated?
5. Might harms outweigh benefits?
   (a) for treatment, (b) for diagnosis

Types of evidence

For questions zero to four any existing, peer reviewed primary studies as well as systematic reviews will be considered. As our preliminary searches came up with very large amounts of evidence from primary studies that could be deemed suitable to answer question five, we will limit the types of evidence included to answer this question as follows: the search for question five part a) will be limited to systematic reviews (of randomised controlled trials or observational studies) and cohort studies investigating short and long term outcomes from ADHD pharmaceutical treatment. The search for question five, part b) will be wider and include any primary studies investigating outcomes after an ADHD diagnosis.

For peer review only - http://bmjopen.bmj.com/site/about/guidelines.xhtml

Search Strategy

Initially, various basic, restricted searches in MEDLINE and Scopus were performed on each of the five pre-determined questions (1 to 5) to uncover some articles relevant to the topic. This initial search was followed by an analysis of key concepts in titles and abstracts as well as index/MeSH terms from key papers. A second full and complex search strategy with the identified keywords and index terms was then developed with the assistance of a research information specialist. This search will be conducted in MEDLINE, Embase, PsychINFO, and the Cochrane Library to locate articles relevant to all five aspects of the framework and the additional primer question (0). For practical reasons this search will be restricted to English language articles only. Databases will be searched from 1979 onwards. Publications from before 1979 will be excluded as their findings would reflect a historic definition of ADHD (hyperkinetic reaction of childhood) in line with the DSM II[39]. These are unlikely to be relevant to our study question. The complete search strategy for MEDLINE can be found in Supplement II. Finally, the search will be supplemented by forward and backward citation searches of all included papers.

Study Selection

After an initial pilot phase to ensure appropriate training for high-level decision making and to test our inclusion/exclusion criteria, all titles and abstracts identified by the database and hand searches will be screened and reviewed for relevance by two researchers independently. Abstrackr (http://abstrackr.cebm.brown.edu), a text mining tool, will be used to help with this initial screening[40 41]. Full-text reviews of all potentially suitable papers will be independently conducted by two researchers, according to the pre-defined inclusion/exclusion criteria. All studies excluded at the full-text screening stage will be reported on with reasons for exclusion provided. At both stages of the screening process (abstract and full-text screening), any discrepancies will be resolved through discussion with the team.

Data Extraction

Data from the final articles to be included in the review will be charted independently into a standardised and piloted template by two researchers. Any uncertainties will be again discussed and resolved by the entire study team. Data will be extracted on the source, eligibility, methods, population characteristics, intervention/exposure, outcomes, results and

For peer review only - http://bmjopen.bmj.com/site/about/guidelines.xhtml

other areas of interest. A template for data extraction is attached (Supplement III) and may be further refined and updated during the review stage.

Presentation of Results

All information regarding the selection of sources will be presented in a flow diagram according to the Preferred Reporting Items for Systematic reviews and Meta-Analyses extension for Scoping Reviews (PRISMA-ScR)[34].

Results from all included studies will be mapped to our pre-determined five questions framework in form of a table as well as in descriptive, narrative form. Quantitative estimates will be included in our synthesis of results where applicable and meaningful. Further, evidence will be categorised by type of article and study type in order to highlight where additional research may be needed to fill current evidence gaps.

PATIENT AND PUBLIC INVOLVEMENT

Neither the protocol nor the proposed scoping review will involve patients or members of the general public.

ETHICS AND DISSEMINATION

Due to the proposed study being a scoping review, there are no ethical or safety considerations to be made. It is planned to disseminate the results from the scoping review through publication in a peer reviewed journal and through conference presentation.

AUTHORS' CONTRIBUTIONS

LK, KB, RT and AB have contributed to the conception and design of the protocol. LK, KB, RT, KM and AB contributed to the establishment of searches. LK drafted the protocol, KB, RT, KM and AB made contributions to the drafting and revising of the article. All authors approved the final version of the protocol for publication and its accuracy and integrity.

ACKNOWLEDGEMENTS

The authors acknowledge the contributions towards the search strategy development and other useful recommendations with regard to literature searching made by Justin Clark,

For peer review only - http://bmjopen.bmj.com/site/about/guidelines.xhtml

Senior Research Information Specialist at the Institute of Evidence-Based Healthcare, Bond University.

Funding statement

This work is supported by Wiser Healthcare, which is funded by the National Health and Medical Research Council (NHMRC) Program Grant 1113532 and CRE Grant 1104136. The funding source has no role in study design, data collection, data analysis, data interpretation, or writing of the report.

Competing interests

None.

References

1. Bell KJL, Doust J, Glasziou P, et al. Recognizing the Potential for Overdiagnosis: Are High-Sensitivity Cardiac Troponin Assays an Example? *Ann Intern Med* 2019;170(4) doi: 10.7326/M18-2645
2. Visser SN, Danielson ML, Bitsko RH, et al. Trends in the parent-report of health care provider-diagnosed and medicated attention-deficit/hyperactivity disorder: United States, 2003-2011. *J Am Acad Child Adolesc Psychiatry* 2014;53(1):34-46 e2. doi: 10.1016/j.jaac.2013.09.001
3. Xu G, Strathearn L, Liu B, et al. Twenty-Year Trends in Diagnosed Attention-Deficit/Hyperactivity Disorder Among US Children and Adolescents, 1997-2016. *JAMA Netw Open* 2018;1(4) doi: 10.1001/jamanetworkopen.2018.1471
4. Rydell M, Lundstrom S, Gillberg C, et al. Has the attention deficit hyperactivity disorder phenotype become more common in children between 2004 and 2014? Trends over 10 years from a Swedish general population sample. *J Child Psychol Psychiatry* 2018;59(8):863-71. doi: 10.1111/jcpp.12882
5. Chien IC, Lin CH, Chou YJ, et al. Prevalence, incidence, and stimulant use of attention-deficit hyperactivity disorder in Taiwan, 1996-2005: a national population-based study. *Soc Psychiatry Psychiatr Epidemiol* 2012;47(12):1885-90. doi: 10.1007/s00127-012-0501-1

For peer review only - http://bmjopen.bmj.com/site/about/guidelines.xhtml

6. Giacobini M, Medin E, Ahnemark E, et al. Prevalence, Patient Characteristics, and Pharmacological Treatment of Children, Adolescents, and Adults Diagnosed With ADHD in Sweden. *J Atten Disord* 2018;22(1):3-13. doi: 10.1177/1087054714554617

7. Coon ER, Quinonez RA, Moyer VA, et al. Overdiagnosis: how our compulsion for diagnosis may be harming children. *Pediatrics* 2014;134(5):1013-23. doi: 10.1542/peds.2014-1778 [published Online First: 2014/10/08]

8. Bruchmueller K, Margraf J, Schneider S. Is ADHD Diagnosed in Accord With Diagnostic Criteria? Overdiagnosis and Influence of Client Gender on Diagnosis. *J Consult Clin Psychol* 2012;80(1) doi: 10.1037/a0026582.supp

9. Batstra L, Frances A. DSM-5 Further Inflates Attention Deficit Hyperactivity Disorder. *J Nerv Ment Dis* 2012;200(6):486-88. doi: 10.1097/NMD.0b013e318257c4b6

10. Batstra L, Hadders-Algra M, Nieweg E, et al. Childhood emotional and behavioral problems: reducing overdiagnosis without risking undertreatment. *Dev Med Child Neurol* 2012;54(6):492-4. doi: 10.1111/j.1469-8749.2011.04176.x [published Online First: 2012/05/11]

11. Barratt A. Overdiagnosis in mammography screening: a 45 year journey from shadowy idea to acknowledged reality. *BMJ* 2015;350:h867. doi: 10.1136/bmj.h867 [published Online First: 2015/03/05]

12. Welch HG, Black WC. Overdiagnosis in cancer. *J Natl Cancer Inst* 2010;102(9):605-13. doi: 10.1093/jnci/djq099 [published Online First: 2010/04/24]

13. Thomas R, Mitchell GK, Batstra L. Attention-deficit/hyperactivity disorder: are we helping or harming? *BMJ* 2013;347:f6172. doi: 10.1136/bmj.f6172 [published Online First: 2013/11/07]

14. Merten EC, Cwik JC, Margraf J, et al. Overdiagnosis of mental disorders in children and adolescents (in developed countries). *Child Adolesc Psychiatry Ment Health* 2017;11:5. doi: 10.1186/s13034-016-0140-5 [published Online First: 2017/01/21]

15. McKeown RE, Holbrook JR, Danielson ML, et al. The Impact of Case Definition on Attention-Deficit/Hyperactivity Disorder Prevalence Estimates in Community-Based Samples of School-Aged Children. *J Am Acad Child Psy* 2015;54(1):53-61. doi: 10.1016/j.jaac.2014.10.014

For peer review only - http://bmjopen.bmj.com/site/about/guidelines.xhtml

16. Brodersen J, Schwartz LM, Heneghan C, et al. Overdiagnosis: what it is and what it isn't. *BMJ Evid Based Med* 2018;23(1):1-3. doi: 10.1136/ebmed-2017-110886 [published Online First: 2018/01/26]

17. Rogers WA, Mintzker Y. Getting clearer on overdiagnosis. *J Eval Clin Pract* 2016;22(4):580-7. doi: 10.1111/jep.12556 [published Online First: 2016/05/07]

18. Safer DJ. Is ADHD Really Increasing in Youth? *J Atten Disord* 2018;22(2):107-15. doi: 10.1177/1087054715586571

19. Morrow R. Influence of relative age on diagnosis and tretament. *CMAJ* 2012;184(7)

20. Danielson ML, Bitsko RH, Ghandour RM, et al. Prevalence of Parent-Reported ADHD Diagnosis and Associated Treatment Among U.S. Children and Adolescents, 2016. *J Clin Child Adolesc Psychol* 2018;47(2):199-212. doi: 10.1080/15374416.2017.1417860 [published Online First: 2018/01/25]

21. Taylor E. Attention deficit hyperactivity disorder: overdiagnosed or diagnoses missed? *Arch Dis Child* 2017;102(4):376-79. doi: 10.1136/archdischild-2016-310487 [published Online First: 2016/11/09]

22. Pathirana T, Clark J, Moynihan R. Mapping the drivers of overdiagnosis to potential solutions. *BMJ* 2017;358:j3879. doi: 10.1136/bmj.j3879

23. Jenniskens K, de Groot JAH, Reitsma JB, et al. Overdiagnosis across medical disciplines: a scoping review. *BMJ Open* 2017;7(12):e018448. doi: 10.1136/bmjopen-2017-018448

24. Thomas R, Sanders S, Doust J, et al. Prevalence of attention-deficit/hyperactivity disorder: a systematic review and meta-analysis. *Pediatrics* 2015;135(4):e994-1001. doi: 10.1542/peds.2014-3482 [published Online First: 2015/03/04]

25. Polanczyk G, de Lima MS, Horta BL, et al. The worldwide prevalence of ADHD: a systematic review and metaregression analysis. *Am J Psychiatry* 2007;164(6):942-8. doi: 10.1176/ajp.2007.164.6.942 [published Online First: 2007/06/02]

26. Polanczyk GV, Willcutt EG, Salum GA, et al. ADHD prevalence estimates across three decades: an updated systematic review and meta-regression analysis. *Int J Epidemiol* 2014;43(2):434-42. doi: 10.1093/ije/dyt261 [published Online First: 2014/01/28]

27. Storebo OJ, Pedersen N, Ramstad E, et al. Methylphenidate for attention deficit hyperactivity disorder (ADHD) in children and adolescents - assessment of adverse events in non-randomised studies. *Cochrane Database Syst Rev* 2018;5:CD012069. doi: 10.1002/14651858.CD012069.pub2

For peer review only

For peer review only - http://bmjopen.bmj.com/site/about/guidelines.xhtml

28. Storebo OJ, Ramstad E, Krogh HB, et al. Methylphenidate for children and adolescents with attention deficit hyperactivity disorder (ADHD). *Cochrane Database Syst Rev* 2015(11):CD009885. doi: 10.1002/14651858.CD009885.pub2

29. Moreira-Maia CR, Massuti R, Tessari L, et al. Are ADHD medications under or over prescribed worldwide?: Protocol for a systematic review and meta-analysis. *Medicine (Baltimore)* 2018;97(24):e10923. doi: 10.1097/MD.0000000000010923 [published Online First: 2018/06/15]

30. Batstra L, Frances A. Holding the line against diagnostic inflation in psychiatry. *Psychother Psychosom* 2012;81(1):5-10. doi: 10.1159/000331565

31. Peters M, Godfrey C, McInerney P, et al. Scoping Reviews. In: Aromataris E, Munn Z, eds. Joanna Briggs Institute Reviewer's Manual. https://reviewersmanual.joannabriggs.org/: The Joanna Briggs Institute 2017.

32. Arksey H, O'Malley L. Scoping studies: towards a methodological framework. *Int J Soc Res Methodol* 2005;8(1):19-32.

33. Levac D, Colquhoun H, O'Brien K. Scoping studies: advancing the methodology. *Implement Sc* 2010;5(1):1-9.

34. Tricco AC, Lillie E, Zarin W, et al. PRISMA Extension for Scoping Reviews (PRISMA-ScR): Checklist and Explanation. *Ann Intern Med* 2018;169(7):467-73. doi: 10.7326/M18-0850

35. Davies L, Petitti DB, Martin L, et al. Defining, Estimating, and Communicating Overdiagnosis in Cancer Screening. *Ann Intern Med* 2018;169(1):36-43. doi: 10.7326/M18-0694

36. Hofmann B. Medicalization and overdiagnosis: different but alike. *Med Health Care Philos* 2016;19(2):253-64. doi: 10.1007/s11019-016-9693-6

37. Carter SM, Degeling C, Doust J, et al. A definition and ethical evaluation of overdiagnosis. *J Med Ethics* 2016;42(11):705-14. doi: 10.1136/medethics-2015-102928 [published Online First: 2016/07/13]

38. Carter SM, Rogers W, Heath I, et al. The challenge of overdiagnosis begins with its definition. *BMJ* 2015;350:h869. doi: 10.1136/bmj.h869 [published Online First: 2015/03/06]

39. American Psychiatric Association. Diagnostic and statistical manual of mental disorders (DSM II). 2nd ed. Washington DC: American Psychiatric Association 1968.

For peer review only - http://bmjopen.bmj.com/site/about/guidelines.xhtml

40. Gates A, Johnson C, Hartling L. Technology-assisted title and abstract screening for systematic reviews: a retrospective evaluation of the Abstrackr machine learning tool. *Syst Rev* 2018;7(1):45. doi: 10.1186/s13643-018-0707-8

41. Rathbone J, Hoffmann T, Glasziou P. Faster title and abstract screening? Evaluating Abstrackr, a semi-automated online screening program for systematic reviewers. *Syst Rev* 2015;4:80. doi: 10.1186/s13643-015-0067-6

For peer review only

For peer review only - http://bmjopen.bmj.com/site/about/guidelines.xhtml

For peer review only

For peer review only - http://bmjopen.bmj.com/site/about/guidelines.xhtml

For peer review only

For peer review only - http://bmjopen.bmj.com/site/about/guidelines.xhtml

SUPPLEMENT I

PREFERRED REPORTING ITEMS FOR SYSTEMATIC REVIEWS AND META-ANALYSES EXTENSION FOR SCOPING REVIEWS (PRISMA-SCR) CHECKLIST

SECTION	ITEM	PRISMA-ScR CHECKLIST ITEM	REPORTED ON PAGE #
**TITLE**			
Title	1	Identify the report as a scoping review.	2
**ABSTRACT**			
Structured summary	2	Provide a structured summary that includes (as applicable): background, objectives, eligibility criteria, sources of evidence, charting methods, results, and conclusions that relate to the review questions and objectives.	2
**INTRODUCTION**			
Rationale	3	Describe the rationale for the review in the context of what is already known. Explain why the review questions/objectives lend themselves to a scoping review approach.	4/5
Objectives	4	Provide an explicit statement of the questions and objectives being addressed with reference to their key elements (e.g., population or participants, concepts, and context) or other relevant key elements used to conceptualize the review questions and/or objectives.	4
**METHODS**			
Protocol and registration	5	Indicate whether a review protocol exists; state if and where it can be accessed (e.g., a Web address); and if available, provide registration information, including the registration number.	N/A for protocol
Eligibility criteria	6	Specify characteristics of the sources of evidence used as eligibility criteria (e.g., years considered, language, and publication status), and provide a rationale.	5/6
Information sources*	7	Describe all information sources in the search (e.g., databases with dates of coverage and contact with authors to identify additional sources), as well as the date the most recent search was executed.	7
Search	8	Present the full electronic search strategy for at least 1 database, including any limits used, such that it could be repeated.	Appendix I, 12
Selection of sources of evidence†	9	State the process for selecting sources of evidence (i.e., screening and eligibility) included in the scoping review.	7
Data charting process‡	10	Describe the methods of charting data from the included sources of evidence (e.g., calibrated forms or forms that have been tested by the team before their use, and whether data charting was done independently or in duplicate) and any processes for obtaining and confirming data from investigators.	7
Data items	11	List and define all variables for which data were sought and any assumptions and simplifications made.	Appendix II, 14

For peer review only

Critical appraisal of individual sources of evidence§	12	If done, provide a rationale for conducting a critical appraisal of included sources of evidence; describe the methods used and how this information was used in any data synthesis (if appropriate).	N/A
Synthesis of results	13	Describe the methods of handling and summarizing the data that were charted.	7
**RESULTS**			
Selection of sources of evidence	14	Give numbers of sources of evidence screened, assessed for eligibility, and included in the review, with reasons for exclusions at each stage, ideally using a flow diagram.	7
Characteristics of sources of evidence	15	For each source of evidence, present characteristics for which data were charted and provide the citations.	N/A for protocol
Critical appraisal within sources of evidence	16	If done, present data on critical appraisal of included sources of evidence (see item 12).	N/A
Results of individual sources of evidence	17	For each included source of evidence, present the relevant data that were charted that relate to the review questions and objectives.	N/A for protocol
Synthesis of results	18	Summarize and/or present the charting results as they relate to the review questions and objectives.	
**DISCUSSION**			
Summary of evidence	19	Summarize the main results (including an overview of concepts, themes, and types of evidence available), link to the review questions and objectives, and consider the relevance to key groups.	N/A for protoco l
Limitations	20	Discuss the limitations of the scoping review process.	N/A for protocol
Conclusions	21	Provide a general interpretation of the results with respect to the review questions and objectives, as well as potential implications and/or next steps.	N/A for protocol
**FUNDING**			
Funding	22	Describe sources of funding for the included sources of evidence, as well as sources of funding for the scoping review. Describe the role of the funders of the scoping review.	8

* Where *sources of evidence* (see second footnote) are compiled from, such as bibliographic databases, social media platforms, and Web sites.

† A more inclusive/heterogeneous term used to account for the different types of evidence or data sources (e.g., quantitative and/or qualitative research, expert opinion, and policy documents) that may be eligible in a scoping review as opposed to only studies. This is not to be confused with *information sources* (see first footnote).

‡ The frameworks by Arksey and O'Malley and Levac and colleagues and the JBI guidance refer to the process of data extraction in a scoping review as data charting.

§ The process of systematically examining research evidence to assess its validity, results, and relevance before using it to inform a decision. This term is used for items 12 and 19 instead of "risk of bias" (which is more applicable to systematic reviews of interventions) to include and acknowledge the various sources of evidence that may be used in a scoping review (e.g., quantitative and/or qualitative research, expert opinion, and policy document).

From: Tricco AC, Lillie E, Zarin W, O'Brien KK, Colquhoun H, Levac D, et al. PRISMA Extension for Scoping Reviews (PRISMA- ScR): Checklist and Explanation. Ann Intern Med;169:467–473. doi: 10.7326/M18-0850

For peer review only

Supplement II

MEDLINE Search Strategy

0. Is ADHD overdiagnosed?	1. Is there potential for increased diagnosis?	2. Is diagnosis actually increased?	3. Are additional cases subclinical or low risk?	4. Are additional cases treated?	5.a) Might harms outweigh benefits for treatment?	5.b) Might harms outweigh benefits for diagnosis?
((exp Attention Deficit Disorder with Hyperactivity/ OR adhd.ti. OR hyperkinesis.ti. OR exp Hyperkinesis/ OR Attention Deficit Hyperactivity Disorder.ti. OR Attention Deficit Disorder with Hyperactivity.ti. OR Hyperkinetic Disorder.ti.)						
AND						
(Child*.tw. OR Child/ OR Adolescen*.tw. OR exp Adolescent/ OR exp Infant/ OR Infan*.tw. OR Minors/ OR p?ediatric*.tw. OR Pediatrics/ OR primary school*.tw. OR school*.tw. OR kindergarten.tw. OR pre-school.tw. OR Pre School.tw. OR elementary school.tw. OR student*.tw. OR secondary school.tw. OR Schools/ OR high school*.tw. OR Child Psychiatry/ OR Adolescent Psychiatry/)						
AND						
(overdiagnos*.mp. OR over diagnos*.mp. OR overtest*.mp. OR over test*.mp. OR exp Medical Overuse/ OR overuse*.mp. OR over use*.mp. OR overdetect*.mp. OR over detect*.mp. OR insignificant disease.mp. OR overtreat*.mp. OR over treat*.mp. OR inconsequential disease.mp. OR overmedical*.mp. OR unnecessary procedure*.mp. OR exp Unnecessary Procedures/ OR pseudodisease.mp. OR pseudo disease.mp. OR "too much medicine".mp. OR	((continuum OR continual* OR continuous* OR dimension* OR categoric* OR spectrum OR subthreshold OR threshold OR full syndrome OR dichotomous OR linear association OR distribution of symptom* OR full symptom*).tw))	(prevalence/ OR prevalen*.ti. OR incidence/ OR inciden*.ti. OR frequency.ti. OR rate.ti. OR definition*.ti. OR diagnos*.ti. OR Diagnosis/ OR phenotype.ti)	(severity.tw. or impair*.tw. or mild.tw. or moderate.tw. or severe.tw. or extreme.tw. or subclinical.tw. or subthreshold.tw)	(treatment*.ti. or exp Therapeutics/ or medication*.ti. or pharma*.ti. or Pharmaceutical Preparations/ or Ritalin.ti. or exp Methylphenidate/ or Central Nervous System Stimulants/ or stimulant*.ti. or drug*.ti. or therapeutics/ or drug therapy/ or therapeutic*.ti. or Methylphenidate.ti. or psychostimulant*.ti. or Dexmethylphenidate.ti. or Dexmethylphenidate Hydrochloride/ or Atomoxetine*.ti. or Atomoxetine Hydrochloride/ or nonpsychostimulant.ti. or exp Amphetamines/ or amphetamine*.ti. or adderrall.ti or antipsychotic*.ti. or Antipsychotic Agents/)		(label*.mp.))
	NOT	AND	AND	AND	AND	NOT
	(Autism.ti. OR Autistic.ti. OR exp Autistic Disorder/ OR exp Autism spectrum	(trend*.tw. OR field trial*.tw. OR follow-up studies/ OR follow-up.tw.	(prevalence/ OR prevalen*.ti. OR incidence/ OR inciden*.ti. OR	(trend*.ti. OR change*.ti. OR variation*.ti. OR vary*.ti. OR	(Treatment Outcome/ or outcome*.tw. OR consequence*.tw. OR impact*.tw.)	(off-label.tw. OR Open-Label.tw.)

For peer review only

nondisease.mp. OR non disease.mp. OR "false positive*".mp. OR overdefinition*.mp. OR over definition*.mp. OR misdiagnos*.mp. or Diagnostic Errors OR variation of care.mp. OR medicali*.mp. OR Medicalization/))	Disorder/)	OR chang*.tw. OR variation*.tw. OR vary*.tw. OR increas*.tw. OR decreas*.tw. OR pattern*.tw. OR expan*.tw.))	frequency.ti. OR rate.ti. OR trend*.ti. OR change*.ti. OR variation*.ti. OR vary*.ti. OR increase*.ti. OR decrease*.ti. OR pattern*.ti. Or expand*.ti.))	increase*.ti. OR decrease*.ti. OR pattern*.ti. Or expand*.ti. OR overprescri*.ti. OR prescri*.ti. OR underprescri*.ti.))	AND<br>(Patient Harm/ or harm*.tw. OR Cost-Benefit Analysis/ OR benefit*.tw. OR cost*.tw. OR Risk/ or risk*.tw. or improv*.tw. or positive.tw. or negative.tw. OR worse.tw. or better.tw. OR adverse.tw. OR effect*.tw.)<br>AND<br>(((meta analysis or "systematic review").pt.) OR (Cohort.tw. OR longitud*.tw. OR observation*.tw. OR follow-up.tw. OR registries/ OR longitudinal studies/)))	
NOT						
((autobiography OR bibliography OR biography OR case reports OR comment OR congress OR consensus development conference, nih OR dataset OR dictionary OR directory OR editorial OR expression of concern OR festschrift OR government document OR guideline OR interactive tutorial OR lecture OR legal case OR legislation OR letter OR news OR newspaper article OR patient education handout OR personal narrative OR portrait OR scientific integrity review OR technical report OR video-audio media).mp. OR webcasts.pt.)						
AND						
limit search to (english language and yr="1979 -Current")						

For peer review only

SUPPLEMENT III

DRAFT DATA COLLECTION ITEMS

Source

- Author
- Title
- Year
- Citation

Eligibility

- Reason for exclusion
- Reason for inclusion
- Answers which question (1-5)

Methods

- Study design
- Study Question/Aim

Population Characteristics

- Number
- Setting
- Age
- Sex
- Country
- Co-morbidity
- Socio-demographics/ethnicity/other

Intervention/Exposure

- Specific Intervention/Exposure
- Comparator
- Number of groups

Outcomes

- Outcomes
- Definition of outcomes/diagnostic criteria used
- Scales/threshold

Results

For peer review only

- Estimate of effect, CIs, p-values

Miscellaneous

- Funding
- Key conclusions
- Comments

Relevant References

For peer review only

BMJ Open

Evidence of potential overdiagnosis and overtreatment of Attention Deficit Hyperactivity Disorder (ADHD) in children and adolescents: protocol for a scoping review

Journal:	*BMJ Open*
Manuscript ID	bmjopen-2019-032327.R1
Article Type:	Protocol
Date Submitted by the Author:	23-Sep-2019
Complete List of Authors:	Kazda, Luise; The University of Sydney, Sydney School of Public Health<br>Bell, Katy; The University of Sydney Faculty of Medicine and Health, School of Public Health; The University of Sydney<br>Thomas, Rae; Bond University, Faculty of Health Sciences and Medicine<br>McGeechan, Kevin; The University of Sydney, School of Public Health;<br>Barratt, Alexandra; University of Sydney, School of Public Health
**Primary Subject Heading**:	Epidemiology
Secondary Subject Heading:	Epidemiology, Paediatrics, Public health, Mental health
Keywords:	EPIDEMIOLOGY, MENTAL HEALTH, PAEDIATRICS, Child & adolescent psychiatry < PSYCHIATRY, PUBLIC HEALTH

SCHOLARONE™
Manuscripts

Evidence of potential overdiagnosis and overtreatment of Attention Deficit Hyperactivity Disorder (ADHD) in children and adolescents: protocol for a scoping review

Luise Kazda[1*], Katy Bell[1], Rae Thomas[2], Kevin McGeechan[1], Alexandra Barratt[1]

[1]Sydney School of Public Health, Faculty of Medicine and Health, The University of Sydney, NSW, Australia

[2]Institute for Evidence-Based Healthcare, Bond University, Gold Coast, Qld, Australia

*Luise Kazda
Room 124, Edward Ford Building (A27), The University of Sydney NSW 2006, Australia
luise.kazda@sydney.edu.au
+61 2 90365360

Word count: 2,329

Key Words: Attention Deficit Hyperactivity Disorder (ADHD), overdiagnosis, overtreatment, children, adolescents

For peer review only

For peer review only - http://bmjopen.bmj.com/site/about/guidelines.xhtml

Title

Evidence of potential overdiagnosis and overtreatment of Attention Deficit Hyperactivity Disorder (ADHD) in children and adolescents: protocol for a scoping review

Abstract

Introduction

Worldwide, ADHD diagnosis rates in children and adolescents have been increasing consistently over the past decades, fuelling a debate about the underlying reasons for this trend. While many hypothesise that a substantial number of these additional cases are overdiagnosed, to date there has been no comprehensive evaluation of evidence for or against this hypothesis. Thus, with this scoping review we aim to synthesise published evidence on the topic, in order to investigate whether existing literature is consistent with the occurrence of overdiagnosis and/or overtreatment of ADHD in children and adolescents.

Methods and Analysis

The proposed scoping review will be conducted in the context of a framework of five questions, developed specifically to identify areas in medicine with the potential for overdiagnosis and overtreatment. The review will adhere to the Joanna Briggs Methodology for Scoping Reviews. We will search MEDLINE, Embase, PsychINFO, and the Cochrane Library electronic databases for primary studies published in English from 1979 onwards. We will also conduct forward and backward citation searches of included articles. Data from studies that meet our pre-defined exclusion and inclusion criteria will be charted into a standardised extraction template with results mapped to our pre-determined five questions framework in form of a table and summarised in narrative form.

Ethics and Dissemination

The proposed study is a scoping review of the existing literature and, as such, does not require ethics approval. We intend to disseminate the results from the scoping review through publication in a peer-reviewed journal and through conference presentations. Further, we will use the findings from our scoping review to inform future research to fill key evidence gaps identified by this review.

For peer review only - http://bmjopen.bmj.com/site/about/guidelines.xhtml

For peer review only

For peer review only - http://bmjopen.bmj.com/site/about/guidelines.xhtml

Strengths and limitations of this study

- First comprehensive synthesis of evidence on the potential for overdiagnosis and overtreatment in ADHD in children and adolescents.
- Broad and systematic search strategy to retrieve all relevant studies published since 1979.
- Compares evidence against established criteria for potential overdiagnosis and overtreatment.
- Includes a critical appraisal of included studies but no formal risk of bias assessment.
- Underdiagnosis and undertreatment may also occur, but are outside the scope of this review.

For peer review only

For peer review only - http://bmjopen.bmj.com/site/about/guidelines.xhtml

Introduction

Review Questions

Our primary study question for this scoping review is: "Does the published literature indicate a potential for overdiagnosis and/or overtreatment of Attention Deficit Hyperactivity Disorder (ADHD) in children and adolescents?" Besides this question, we will consider two further, secondary questions in our review: "When mapped against an existing framework for identifying potential overdiagnosis[1], are there any gaps in the current evidence?" and "How does the framework[1] perform in terms of applicability and usability for identifying potential overdiagnosis of a mental health condition?"

Background

With steadily increasing prevalence rates of ADHD throughout the developed world[2-6], there is growing debate about whether this trend is due to an actual increase in prevalence, better detection and diagnosis, misdiagnosis, or overdiagnosis[7-10]. Whilst the evidence for overdiagnosis in many other conditions (especially in screen detected cancers) is increasingly recognised[1 11 12], the evidence for overdiagnosis of ADHD, a non-cancer condition where overdiagnosis is widely thought to occur, has not yet been comprehensively evaluated[13]. Quantifying the potential for overdiagnosis is only just emerging as a field of interest in both paediatric and mental health research[7 14].

The potential overdiagnosis of ADHD could be caused by three main drivers. Firstly, it can arise due to the problem of overdefinition[15], that is, lowering the threshold for a disease, by expanding the disease definition to include people with ambiguous or very mild symptoms without evidence that doing so improves patients' health overall and in the longer term[16 17]. Secondly, overdiagnosis may also be caused by overdetection[4 18] (e.g., screening children at young ages for behaviour problems) and, thirdly, by the medicalisation of some behaviour patterns (e.g. those typical of relatively younger school children)[19]. Other factors may also have played a part. These include formal changes to the diagnostic threshold (DSM-5 vs DSM-IV), pharmaceutical industry influence[13] and health and social service drivers (e.g. access to resources linked to a diagnosis)[10].

Although the prevalence rates of ADHD seem to have risen substantially in the past few years, most cases continue to be reported as mild to moderate forms (87% in 2007, 84% in 2011 and

For peer review only - http://bmjopen.bmj.com/site/about/guidelines.xhtml

86% in 2016 of all reported cases in a large US based survey)[2 20] and it has been argued that many of those cases may represent overdiagnosis. Should this be the case, these children may not experience a net benefit from an ADHD diagnosis and subsequent treatments, but may be harmed[13].

While correctly diagnosing and treating ADHD has many potential benefits[21] the harms from overdiagnosing and overtreating ADHD are significant and costly on multiple levels. Not only may the individual child experience negative physical and psychosocial effects, their families may also experience psychosocial and financial burdens. Overdiagnosis and overtreatment also results in financial and opportunity costs to the health system, and to society at large[7]. It has also been pointed out that the resulting potential overuse of healthcare resources contributes to the simultaneous underuse of said resources[22]; e.g. by depriving underdiagnosed and undertreated groups of children who would largely benefit from ADHD treatment and timely access to diagnostic and treatment services[23]. Whilst also addressing the twin issues of underdiagnosis and undertreatment is beyond the scope of this proposed review, we see our work as a starting point for a broader discussion around the principles of “right care” where resources need to be re-allocated to where they are most needed and effective[22].

Rationale

In summary, while there are increasing concerns about the potential for overdiagnosis of ADHD in children and adolescents, there is scant evidence quantifying the problem. Consequently, a systematic review of the literature to quantify ADHD overdiagnosis is not possible due to the current lack of synthesisable evidence in this field. The rationale for this broader scoping review then, is to use a recently developed framework of questions[1] to systematically determine if the existing literature indicates a potential for overdiagnosis and overtreatment in ADHD. We hypothesize that ADHD fulfils these pre-determined criteria for potential overdiagnosis. A secondary aim is to further examine and highlight any gaps in the current evidence that may prevent us from determining whether or not ADHD is overdiagnosed and overtreated. This aim will be especially helpful in guiding subsequent research in this area. A further secondary aim of the study is to rigorously test the existing 5-questions-framework[1] for its applicability and usability in another area, namely paediatric mental health.

For peer review only - http://bmjopen.bmj.com/site/about/guidelines.xhtml

Preliminary searches of the literature were conducted in March and April 2019 to determine if any previous scoping or systematic reviews had been conducted or protocols submitted which aimed to summarise existing evidence on overdiagnosis and overtreatment in ADHD. Databases searched were the Cochrane Database of Systematic Reviews, PROSPERO, MEDLINE, Scopus and the JBI Database of Systematic Reviews and Implementation Reports. Two scoping reviews on overdiagnosis in health care were found but neither review was focused on overdiagnosis and overtreatment of ADHD. One review covered the drivers of overdiagnosis and potential solutions[22], while the other addressed overdiagnosis across different medical disciplines[24 25]). Whilst a number of systematic reviews have been conducted on ADHD, these have been restricted to prevalence[26-28] or treatment options[29 30]. We also identified one systematic review on over- or under-prescribing of ADHD medication that is currently underway with a published protocol[31]. Literary reviews and commentaries on overdiagnosis in mental health, including ADHD have been published[13 21 32]. However, we found no reviews that have been conducted or are in preparation, that systematically gather and analyse all available evidence to allow a comprehensive assessment on the potential for overdiagnosis of ADHD.

METHODS AND ANALYSIS

The proposed review will follow the Joanna Briggs Methodology for Scoping Reviews[33] which is based on and extends the work of Arksey and O'Malley[34] as well as that of Levac and colleagues[35]. The scoping review will also adhere to the Preferred Reporting Items for Systematic Reviews and Meta-Analyses Extension for Scoping Reviews (PRISMA-ScR)[36] (Supplement I). This approach was chosen to document the review process as clearly and rigorously as possible. The timeframe for undertaking of the review is from 13th June 2019 (date that the final search strategy was run in all included databases) until 31st December (anticipated completion date of the review).

Inclusion Criteria

<u>Participants</u>

The scoping review will include studies whose main focus is on children and adolescents under the age of 18, who have either been clinically diagnosed or identified by parent – teacher – or self-report as having behavioural symptoms of ADHD. Articles that have a clear emphasis on

For peer review only - http://bmjopen.bmj.com/site/about/guidelines.xhtml

adult ADHD will be excluded unless they are longitudinal follow-up studies where participants were determined to have ADHD in childhood and were then followed through into adulthood. Moreover, studies with other health issues or disabilities as the primary focus will also be excluded. However, studies considering any other comorbidities as a secondary diagnosis will be eligible for inclusion if the focus of the study is clearly on ADHD (outcomes on any other disorders will not be reported on or included in the analysis).

Concept

The two core concepts to be examined by this scoping review are "overdiagnosis of ADHD" and "overtreatment of ADHD". Debate over the definition of overdiagnosis is ongoing[1 16 17 37 38], especially in non-cancer contexts. We define overdiagnosis as occurring where a person is correctly diagnosed (according to contemporary professional standards) with a condition but the net effect of the diagnosis for the individual concerned is unfavourable (i.e. when consideration is given to the balance of potential harms and benefits)[1 39]. The resulting overtreatment can then be defined as receiving treatment following an overdiagnosis (or among overdiagnosed individuals)[16 40]. It is important to note that overtreatment can occur as a result of overdiagnosis but also without it, due to other drivers[16]. In this scoping review we will only consider pharmaceutical treatment options for ADHD in terms of overtreatment.

Context

The study will be conducted in the context of a previously published and tested framework of five questions that identify characteristics consistent with the occurrence of overdiagnosis and overtreatment[1]. It is not limited to any geographic areas or settings. The five questions were developed by experts in the field of overdiagnosis as a guide to identifying areas in medicine where overdiagnosis and -treatment may be occurring. Additionally, an overarching primer question will be included to avoid missing evidence. Hence, the questions by which the search will be guided and to which the evidence will be mapped are:

0. Is ADHD overdiagnosed in children and adolescents?
1. Is there potential for increased diagnosis?
2. Is diagnosis actually increased?
3. Are additional cases subclinical or low risk?
4. Are additional cases treated?

For peer review only - http://bmjopen.bmj.com/site/about/guidelines.xhtml

5. Might harms outweigh benefits?
   (a) for treatment, (b) for diagnosis

Types of evidence

For questions zero to four any existing, peer reviewed primary studies as well as systematic reviews will be considered. As our preliminary searches came up with very large amounts of evidence from primary studies that could be deemed suitable to answer question five, we will limit the types of evidence included to answer this question as follows: the search for question five part a) will be limited to systematic reviews (of randomised controlled trials or observational studies) and cohort studies investigating short and long term outcomes from ADHD pharmaceutical treatment. The search for question five, part b) will be wider and include any primary studies investigating outcomes after an ADHD diagnosis.

Search Strategy

Initially, various basic, restricted searches in MEDLINE and Scopus were performed on each of the five pre-determined questions (1 to 5) to uncover some articles relevant to the topic. This initial search was followed by an analysis of key concepts in titles and abstracts as well as index/MeSH terms from key papers. Moreover, we reviewed published search strategies from reviews on similar topics to identify key words related to our study aims[24 25]. A second full and complex search strategy with the identified keywords and index terms was then developed with the assistance of a research information specialist. This search will be conducted in MEDLINE, Embase, PsychINFO, and the Cochrane Library to locate articles relevant to all five aspects of the framework and the additional primer question (0). For practical reasons this search will be restricted to English language articles only. Databases will be searched from 1979 onwards. Publications from before 1979 will be excluded as their findings would reflect a historic definition of ADHD (hyperkinetic reaction of childhood) in line with the DSM II[41]. These are unlikely to be relevant to our study question. The complete search strategy for MEDLINE can be found in Supplement II. Finally, the search will be supplemented by forward and backward citation searches of all included papers.

Study Selection

After an initial pilot phase to ensure appropriate training for high-level decision making and to test our inclusion/exclusion criteria, all titles and abstracts identified by the database and

For peer review only - http://bmjopen.bmj.com/site/about/guidelines.xhtml

hand searches will be screened and reviewed for relevance by two researchers independently. Abstrackr (http://abstrackr.cebm.brown.edu), a text mining tool, will be used to help with this initial screening[42 43]. Full-text reviews of all potentially suitable papers will be independently conducted by two researchers, according to the pre-defined inclusion/exclusion criteria. All studies excluded at the full-text screening stage will be reported on with reasons for exclusion provided. At both stages of the screening process (abstract and full-text screening), any discrepancies will be resolved through discussion with the team.

For peer review only

For peer review only - http://bmjopen.bmj.com/site/about/guidelines.xhtml

Data Extraction

Data from the final articles to be included in the review will be charted independently into a standardised and piloted template by two researchers. Any uncertainties will be again discussed and resolved by the entire study team. Data will be extracted on the source, eligibility, methods, population characteristics, intervention/exposure, outcomes, results and other areas of interest. A template for data extraction is attached (Supplement III) and may be further refined and updated during the review stage. As part of the data extraction process all included studies will undergo a basic critical appraisal using the Joanna Briggs Institute Critical Appraisal Tools for the relevant study type (https://joannabriggs.org/critical_appraisal_tools).

Presentation of Results

All information regarding the selection of sources will be presented in a flow diagram according to the Preferred Reporting Items for Systematic reviews and Meta-Analyses extension for Scoping Reviews (PRISMA-ScR)[36].

Results from all included studies will be mapped to our pre-determined five questions framework in form of a table as well as in descriptive, narrative form. This results table will include summary information from the conducted critical appraisals. Estimates from quantitative studies will be included in a summary table but will not be meta-analysed as we expect results to be too heterogeneous to allow for meaningful synthesis. Further, evidence will be categorised by type of article and study type in order to highlight where additional research may be needed to fill current evidence gaps.

Patient and Public Involvement

Neither the protocol nor the proposed scoping review will involve patients or members of the general public.

ETHICS AND DISSEMINATION

Due to the proposed study being a scoping review, there are no ethical or safety considerations to be made. It is planned to disseminate the results from the scoping review through publication in a peer reviewed journal and through conference presentation.

AUTHORS' CONTRIBUTIONS

For peer review only - http://bmjopen.bmj.com/site/about/guidelines.xhtml

LK, KB, RT and AB have contributed to the conception and design of the protocol. LK, KB, RT, KM and AB contributed to the establishment of searches. LK drafted the protocol, KB, RT, KM and AB made contributions to the drafting and revising of the article. All authors approved the final version of the protocol for publication and its accuracy and integrity.

For peer review only

For peer review only - http://bmjopen.bmj.com/site/about/guidelines.xhtml

ACKNOWLEDGEMENTS

The authors acknowledge the contributions towards the search strategy development and other useful recommendations with regard to literature searching made by Justin Clark, Senior Research Information Specialist at the Institute of Evidence-Based Healthcare, Bond University.

FUNDING STATEMENT

This work is supported by Wiser Healthcare, which is funded by the National Health and Medical Research Council (NHMRC) Program Grant 1113532 and CRE Grant 1104136. The funding source has no role in study design, data collection, data analysis, data interpretation, or writing of the report.

For peer review only

COMPETING INTERESTS

None.

REFERENCES

1. Bell KJL, Doust J, Glasziou P, et al. Recognizing the Potential for Overdiagnosis: Are High-Sensitivity Cardiac Troponin Assays an Example? *Ann Intern Med* 2019;170(4) doi: 10.7326/M18-2645
2. Visser SN, Danielson ML, Bitsko RH, et al. Trends in the parent-report of health care provider-diagnosed and medicated attention-deficit/hyperactivity disorder: United States, 2003-2011. *J Am Acad Child Adolesc Psychiatry* 2014;53(1):34-46 e2. doi: 10.1016/j.jaac.2013.09.001
3. Xu G, Strathearn L, Liu B, et al. Twenty-Year Trends in Diagnosed Attention-Deficit/Hyperactivity Disorder Among US Children and Adolescents, 1997-2016. *JAMA Netw Open* 2018;1(4) doi: 10.1001/jamanetworkopen.2018.1471
4. Rydell M, Lundstrom S, Gillberg C, et al. Has the attention deficit hyperactivity disorder phenotype become more common in children between 2004 and 2014? Trends over 10 years from a Swedish general population sample. *J Child Psychol Psychiatry* 2018;59(8):863-71. doi: 10.1111/jcpp.12882

For peer review only - http://bmjopen.bmj.com/site/about/guidelines.xhtml

5. Chien IC, Lin CH, Chou YJ, et al. Prevalence, incidence, and stimulant use of attention-deficit hyperactivity disorder in Taiwan, 1996-2005: a national population-based study. *Soc Psychiatry Psychiatr Epidemiol* 2012;47(12):1885-90. doi: 10.1007/s00127-012-0501-1

6. Giacobini M, Medin E, Ahnemark E, et al. Prevalence, Patient Characteristics, and Pharmacological Treatment of Children, Adolescents, and Adults Diagnosed With ADHD in Sweden. *J Atten Disord* 2018;22(1):3-13. doi: 10.1177/1087054714554617

7. Coon ER, Quinonez RA, Moyer VA, et al. Overdiagnosis: how our compulsion for diagnosis may be harming children. *Pediatrics* 2014;134(5):1013-23. doi: 10.1542/peds.2014-1778 [published Online First: 2014/10/08]

8. Bruchmueller K, Margraf J, Schneider S. Is ADHD Diagnosed in Accord With Diagnostic Criteria? Overdiagnosis and Influence of Client Gender on Diagnosis. *J Consult Clin Psychol* 2012;80(1) doi: 10.1037/a0026582.supp

9. Batstra L, Frances A. DSM-5 Further Inflates Attention Deficit Hyperactivity Disorder. *J Nerv Ment Dis* 2012;200(6):486-88. doi: 10.1097/NMD.0b013e318257c4b6

10. Batstra L, Hadders-Algra M, Nieweg E, et al. Childhood emotional and behavioral problems: reducing overdiagnosis without risking undertreatment. *Dev Med Child Neurol* 2012;54(6):492-4. doi: 10.1111/j.1469-8749.2011.04176.x [published Online First: 2012/05/11]

11. Barratt A. Overdiagnosis in mammography screening: a 45 year journey from shadowy idea to acknowledged reality. *BMJ* 2015;350:h867. doi: 10.1136/bmj.h867 [published Online First: 2015/03/05]

12. Welch HG, Black WC. Overdiagnosis in cancer. *J Natl Cancer Inst* 2010;102(9):605-13. doi: 10.1093/jnci/djq099 [published Online First: 2010/04/24]

13. Thomas R, Mitchell GK, Batstra L. Attention-deficit/hyperactivity disorder: are we helping or harming? *BMJ* 2013;347:f6172. doi: 10.1136/bmj.f6172 [published Online First: 2013/11/07]

14. Merten EC, Cwik JC, Margraf J, et al. Overdiagnosis of mental disorders in children and adolescents (in developed countries). *Child Adolesc Psychiatry Ment Health* 2017;11:5. doi: 10.1186/s13034-016-0140-5 [published Online First: 2017/01/21]

15. McKeown RE, Holbrook JR, Danielson ML, et al. The Impact of Case Definition on Attention-Deficit/Hyperactivity Disorder Prevalence Estimates in Community-Based

For peer review only

For peer review only - http://bmjopen.bmj.com/site/about/guidelines.xhtml

Samples of School-Aged Children. *J Am Acad Child Psy* 2015;54(1):53-61. doi: 10.1016/j.jaac.2014.10.014

16. Brodersen J, Schwartz LM, Heneghan C, et al. Overdiagnosis: what it is and what it isn't. *BMJ Evid Based Med* 2018;23(1):1-3. doi: 10.1136/ebmed-2017-110886 [published Online First: 2018/01/26]
17. Rogers WA, Mintzker Y. Getting clearer on overdiagnosis. *J Eval Clin Pract* 2016;22(4):580-7. doi: 10.1111/jep.12556 [published Online First: 2016/05/07]
18. Safer DJ. Is ADHD Really Increasing in Youth? *J Atten Disord* 2018;22(2):107-15. doi: 10.1177/1087054715586571
19. Morrow R. Influence of relative age on diagnosis and tretament. *CMAJ* 2012;184(7)
20. Danielson ML, Bitsko RH, Ghandour RM, et al. Prevalence of Parent-Reported ADHD Diagnosis and Associated Treatment Among U.S. Children and Adolescents, 2016. *J Clin Child Adolesc Psychol* 2018;47(2):199-212. doi: 10.1080/15374416.2017.1417860 [published Online First: 2018/01/25]
21. Taylor E. Attention deficit hyperactivity disorder: overdiagnosed or diagnoses missed? *Arch Dis Child* 2017;102(4):376-79. doi: 10.1136/archdischild-2016-310487 [published Online First: 2016/11/09]
22. Elshaug AG, Rosenthal MB, Lavis JN, et al. Levers for addressing medical underuse and overuse: achieving high-value health care. *The Lancet* 2017;390(10090):191-202. doi: 10.1016/s0140-6736(16)32586-7
23. Coker TR, Elliott MN, Toomey SL, et al. Racial and Ethnic Disparities in ADHD Diagnosis and Treatment. *Pediatrics* 2016;138(3) doi: 10.1542/peds.2016-0407
24. Pathirana T, Clark J, Moynihan R. Mapping the drivers of overdiagnosis to potential solutions. *BMJ* 2017;358:j3879. doi: 10.1136/bmj.j3879
25. Jenniskens K, de Groot JAH, Reitsma JB, et al. Overdiagnosis across medical disciplines: a scoping review. *BMJ Open* 2017;7(12):e018448. doi: 10.1136/bmjopen-2017-018448
26. Thomas R, Sanders S, Doust J, et al. Prevalence of attention-deficit/hyperactivity disorder: a systematic review and meta-analysis. *Pediatrics* 2015;135(4):e994-1001. doi: 10.1542/peds.2014-3482 [published Online First: 2015/03/04]
27. Polanczyk G, de Lima MS, Horta BL, et al. The worldwide prevalence of ADHD: a systematic review and metaregression analysis. *Am J Psychiatry* 2007;164(6):942-8. doi: 10.1176/ajp.2007.164.6.942 [published Online First: 2007/06/02]

For peer review only

For peer review only - http://bmjopen.bmj.com/site/about/guidelines.xhtml

28. Polanczyk GV, Willcutt EG, Salum GA, et al. ADHD prevalence estimates across three decades: an updated systematic review and meta-regression analysis. *Int J Epidemiol* 2014;43(2):434-42. doi: 10.1093/ije/dyt261 [published Online First: 2014/01/28]

29. Storebo OJ, Pedersen N, Ramstad E, et al. Methylphenidate for attention deficit hyperactivity disorder (ADHD) in children and adolescents - assessment of adverse events in non-randomised studies. *Cochrane Database Syst Rev* 2018;5:CD012069. doi: 10.1002/14651858.CD012069.pub2

30. Storebo OJ, Ramstad E, Krogh HB, et al. Methylphenidate for children and adolescents with attention deficit hyperactivity disorder (ADHD). *Cochrane Database Syst Rev* 2015(11):CD009885. doi: 10.1002/14651858.CD009885.pub2

31. Moreira-Maia CR, Massuti R, Tessari L, et al. Are ADHD medications under or over prescribed worldwide?: Protocol for a systematic review and meta-analysis. *Medicine (Baltimore)* 2018;97(24):e10923. doi: 10.1097/MD.0000000000010923 [published Online First: 2018/06/15]

32. Batstra L, Frances A. Holding the line against diagnostic inflation in psychiatry. *Psychother Psychosom* 2012;81(1):5-10. doi: 10.1159/000331565

33. Peters M, Godfrey C, McInerney P, et al. Scoping Reviews. In: Aromataris E, Munn Z, eds. Joanna Briggs Institute Reviewer's Manual. https://reviewersmanual.joannabriggs.org/: The Joanna Briggs Institute 2017.

34. Arksey H, O'Malley L. Scoping studies: towards a methodological framework. *Int J Soc Res Methodol* 2005;8(1):19-32.

35. Levac D, Colquhoun H, O'Brien K. Scoping studies: advancing the methodology. *Implement Sc* 2010;5(1):1-9.

36. Tricco AC, Lillie E, Zarin W, et al. PRISMA Extension for Scoping Reviews (PRISMA-ScR): Checklist and Explanation. *Ann Intern Med* 2018;169(7):467-73. doi: 10.7326/M18-0850

37. Davies L, Petitti DB, Martin L, et al. Defining, Estimating, and Communicating Overdiagnosis in Cancer Screening. *Ann Intern Med* 2018;169(1):36-43. doi: 10.7326/M18-0694

38. Hofmann B. Medicalization and overdiagnosis: different but alike. *Med Health Care Philos* 2016;19(2):253-64. doi: 10.1007/s11019-016-9693-6

For peer review only - http://bmjopen.bmj.com/site/about/guidelines.xhtml

39. Carter SM, Degeling C, Doust J, et al. A definition and ethical evaluation of overdiagnosis. *J Med Ethics* 2016;42(11):705-14. doi: 10.1136/medethics-2015-102928 [published Online First: 2016/07/13]

40. Carter SM, Rogers W, Heath I, et al. The challenge of overdiagnosis begins with its definition. *BMJ* 2015;350:h869. doi: 10.1136/bmj.h869 [published Online First: 2015/03/06]

41. American Psychiatric Association. Diagnostic and statistical manual of mental disorders (DSM II). 2nd ed. Washington DC: American Psychiatric Association 1968.

42. Gates A, Johnson C, Hartling L. Technology-assisted title and abstract screening for systematic reviews: a retrospective evaluation of the Abstrackr machine learning tool. *Syst Rev* 2018;7(1):45. doi: 10.1186/s13643-018-0707-8

43. Rathbone J, Hoffmann T, Glasziou P. Faster title and abstract screening? Evaluating Abstrackr, a semi-automated online screening program for systematic reviewers. *Syst Rev* 2015;4:80. doi: 10.1186/s13643-015-0067-6

For peer review only

For peer review only - http://bmjopen.bmj.com/site/about/guidelines.xhtml

SUPPLEMENT I

PREFERRED REPORTING ITEMS FOR SYSTEMATIC REVIEWS AND META-ANALYSES EXTENSION FOR SCOPING REVIEWS (PRISMA-SCR) CHECKLIST

SECTION	ITEM	PRISMA-ScR CHECKLIST ITEM	REPORTED ON PAGE #
**TITLE**			
Title	1	Identify the report as a scoping review.	2
**ABSTRACT**			
Structured summary	2	Provide a structured summary that includes (as applicable): background, objectives, eligibility criteria, sources of evidence, charting methods, results, and conclusions that relate to the review questions and objectives.	2
**INTRODUCTION**			
Rationale	3	Describe the rationale for the review in the context of what is already known. Explain why the review questions/objectives lend themselves to a scoping review approach.	4/5
Objectives	4	Provide an explicit statement of the questions and objectives being addressed with reference to their key elements (e.g., population or participants, concepts, and context) or other relevant key elements used to conceptualize the review questions and/or objectives.	4
**METHODS**			
Protocol and registration	5	Indicate whether a review protocol exists; state if and where it can be accessed (e.g., a Web address); and if available, provide registration information, including the registration number.	N/A for protocol
Eligibility criteria	6	Specify characteristics of the sources of evidence used as eligibility criteria (e.g., years considered, language, and publication status), and provide a rationale.	5/6
Information sources*	7	Describe all information sources in the search (e.g., databases with dates of coverage and contact with authors to identify additional sources), as well as the date the most recent search was executed.	7
Search	8	Present the full electronic search strategy for at least 1 database, including any limits used, such that it could be repeated.	Appendix I, 12
Selection of sources of evidence†	9	State the process for selecting sources of evidence (i.e., screening and eligibility) included in the scoping review.	7
Data charting process‡	10	Describe the methods of charting data from the included sources of evidence (e.g., calibrated forms or forms that have been tested by the team before their use, and whether data charting was done independently or in duplicate) and any processes for obtaining and confirming data from investigators.	7
Data items	11	List and define all variables for which data were sought and any assumptions and simplifications made.	Appendix II, 14
Critical appraisal of individual sources of evidence§	12	If done, provide a rationale for conducting a critical appraisal of included sources of evidence; describe the methods used and how this information was used in any data synthesis (if appropriate).	N/A
Synthesis of results	13	Describe the methods of handling and summarizing the data that were charted.	7
**RESULTS**			

For peer review only

Selection of sources of evidence	14	Give numbers of sources of evidence screened, assessed for eligibility, and included in the review, with reasons for exclusions at each stage, ideally using a flow diagram.	7
Characteristics of sources of evidence	15	For each source of evidence, present characteristics for which data were charted and provide the citations.	N/A for protocol
Critical appraisal within sources of evidence	16	If done, present data on critical appraisal of included sources of evidence (see item 12).	N/A
Results of individual sources of evidence	17	For each included source of evidence, present the relevant data that were charted that relate to the review questions and objectives.	N/A for protocol
Synthesis of results	18	Summarize and/or present the charting results as they relate to the review questions and objectives.	
**DISCUSSION**			
Summary of evidence	19	Summarize the main results (including an overview of concepts, themes, and types of evidence available), link to the review questions and objectives, and consider the relevance to key groups.	N/A for protocol
Limitations	20	Discuss the limitations of the scoping review process.	N/A for protocol
Conclusions	21	Provide a general interpretation of the results with respect to the review questions and objectives, as well as potential implications and/or next steps.	N/A for protocol
**FUNDING**			
Funding	22	Describe sources of funding for the included sources of evidence, as well as sources of funding for the scoping review. Describe the role of the funders of the scoping review.	8

* Where *sources of evidence* (see second footnote) are compiled from, such as bibliographic databases, social media platforms, and Web sites.
† A more inclusive/heterogeneous term used to account for the different types of evidence or data sources (e.g., quantitative and/or qualitative research, expert opinion, and policy documents) that may be eligible in a scoping review as opposed to only studies. This is not to be confused with *information sources* (see first footnote).
‡ The frameworks by Arksey and O'Malley and Levac and colleagues and the JBI guidance refer to the process of data extraction in a scoping review as data charting.
§ The process of systematically examining research evidence to assess its validity, results, and relevance before using it to inform a decision. This term is used for items 12 and 19 instead of "risk of bias" (which is more applicable to systematic reviews of interventions) to include and acknowledge the various sources of evidence that may be used in a scoping review (e.g., quantitative and/or qualitative research, expert opinion, and policy document).

From: Tricco AC, Lillie E, Zarin W, O'Brien KK, Colquhoun H, Levac D, et al. PRISMA Extension for Scoping Reviews (PRISMA- ScR): Checklist and Explanation. Ann Intern Med;169:467–473. doi: 10.7326/M18-0850

For peer review only

SUPPLEMENT II

MEDLINE SEARCH STRATEGY

<table>
<tr><th>0. Is ADHD overdiagnosed?</th><th>1. Is there potential for increased diagnosis?</th><th>2. Is diagnosis actually increased?</th><th>3. Are additional cases subclinical or low risk?</th><th>4. Are additional cases treated?</th><th>5.a) Might harms outweigh benefits for treatment?</th><th>5.b) Might harms outweigh benefits for diagnosis?</th></tr>
<tr><td colspan="7">((exp Attention Deficit Disorder with Hyperactivity/ OR adhd.ti. OR hyperkinesis.ti. OR exp Hyperkinesis/ OR Attention Deficit Hyperactivity Disorder.ti. OR Attention Deficit Disorder with Hyperactivity.ti. OR Hyperkinetic Disorder.ti.)</td></tr>
<tr><td colspan="7">AND</td></tr>
<tr><td colspan="7">(Child*.tw. OR Child/ OR Adolescen*.tw. OR exp Adolescent/ OR exp Infant/ OR Infan*.tw. OR Minors/ OR p?ediatric*.tw. OR Pediatrics/ OR primary school*.tw. OR school*.tw. OR kindergarten.tw. OR pre-school.tw. OR Pre School.tw. OR elementary school.tw. OR student*.tw. OR secondary school.tw. OR Schools/ OR high school*.tw. OR Child Psychiatry/ OR Adolescent Psychiatry/)</td></tr>
<tr><td colspan="7">AND</td></tr>
<tr><td rowspan="4">(overdiagnos*.mp. OR over diagnos*.mp. OR overtest*.mp. OR over test*.mp. OR exp Medical Overuse/ OR overuse*.mp. OR over use*.mp. OR overdetect*.mp. OR over detect*.mp. OR insignificant disease.mp. OR overtreat*.mp. OR over treat*.mp. OR inconsequential disease.mp. OR overmedical*.mp. OR unnecessary procedure*.mp. OR exp Unnecessary Procedures/ OR pseudodisease.mp. OR pseudo disease.mp. OR "too much medicine".mp. OR nondisease.mp. OR non</td><td>((continuum OR continual* OR continuous* OR dimension* OR categoric* OR spectrum OR subthreshold OR threshold OR full syndrome OR dichotomous OR linear association OR distribution of symptom* OR full symptom*).tw))</td><td>(prevalence/ OR prevalen*.ti. OR incidence/ OR inciden*.ti. OR frequency.ti. OR rate.ti. OR definition*.ti. OR diagnos*.ti. OR Diagnosis/ OR phenotype.ti)</td><td>(severity.tw. or impair*.tw. or mild.tw. or moderate.tw. or severe.tw. or extreme.tw. or subclinical.tw. or subthreshold.tw)</td><td colspan="2">(treatment*.ti. or exp Therapeutics/ or medication*.ti. or pharma*.ti. or Pharmaceutical Preparations/ or Ritalin.ti. or exp Methylphenidate/ or Central Nervous System Stimulants/ or stimulant*.ti. or drug*.ti. or therapeutics/ or drug therapy/ or therapeutic*.ti. or Methylphenidate.ti. or psychostimulant*.ti. or Dexmethylphenidate.ti. or Dexmethylphenidate Hydrochloride/ or Atomoxetine*.ti. or Atomoxetine Hydrochloride/ or nonpsychostimulant.ti. or exp Amphetamines/ or amphetamine*.ti. or adderrall.ti or antipsychotic*.ti. or Antipsychotic Agents/)</td><td>(label*.mp.))</td></tr>
<tr><td>NOT</td><td>AND</td><td>AND</td><td>AND</td><td>AND</td><td>NOT</td></tr>
<tr><td rowspan="2">(Autism.ti. OR Autistic.ti. OR exp Autistic Disorder/ OR exp Autism spectrum Disorder/)</td><td rowspan="2">(trend*.tw. OR field trial*.tw. OR follow-up studies/ OR follow-up.tw. OR chang*.tw. OR</td><td rowspan="2">(prevalence/ OR prevalen*.ti. OR incidence/ OR inciden*.ti. OR frequency.ti. OR</td><td rowspan="2">(trend*.ti. OR change*.ti. OR variation*.ti. OR vary*.ti. OR increase*.ti. OR</td><td>(Treatment Outcome/ or outcome*.tw. OR consequence*.tw. OR impact*.tw.)</td><td rowspan="2">(off-label.tw. OR Open-Label.tw.)</td></tr>
<tr><td>AND</td></tr>
</table>

For peer review only

<table>
<tr>
<td rowspan="3">disease.mp. OR "false positive*".mp. OR overdefinition*.mp. OR over definition*.mp. OR misdiagnos*.mp. or Diagnostic Errors OR variation of care.mp. OR medicali*.mp. OR Medicalization/))</td>
<td rowspan="3"></td>
<td rowspan="3">variation*.tw. OR vary*.tw. OR increas*.tw. OR decreas*.tw. OR pattern*.tw. OR expan*.tw.))</td>
<td rowspan="3">rate.ti. OR trend*.ti. OR change*.ti. OR variation*.ti. OR vary*.ti. OR increase*.ti. OR decrease*.ti. OR pattern*.ti. Or expand*.ti.))</td>
<td rowspan="3">decrease*.ti. OR pattern*.ti. Or expand*.ti. OR overprescri*.ti. OR prescri*.ti. OR underprescri*.ti.))</td>
<td>(Patient Harm/ or harm*.tw. OR Cost-Benefit Analysis/ OR benefit*.tw. OR cost*.tw. OR Risk/ or risk*.tw. or improv*.tw. or positive.tw. or negative.tw. OR worse.tw. or better.tw. OR adverse.tw. OR effect*.tw.)</td>
<td rowspan="3"></td>
</tr>
<tr>
<td>AND</td>
</tr>
<tr>
<td>(((meta analysis or "systematic review").pt.) OR (Cohort.tw. OR longitud*.tw. OR observation*.tw. OR follow-up.tw. OR registries/ OR longitudinal studies/)))</td>
</tr>
<tr>
<td colspan="7">NOT</td>
</tr>
<tr>
<td colspan="7">((autobiography OR bibliography OR biography OR case reports OR comment OR congress OR consensus development conference, nih OR dataset OR dictionary OR directory OR editorial OR expression of concern OR festschrift OR government document OR guideline OR interactive tutorial OR lecture OR legal case OR legislation OR letter OR news OR newspaper article OR patient education handout OR personal narrative OR portrait OR scientific integrity review OR technical report OR video-audio media).mp. OR webcasts.pt.)</td>
</tr>
<tr>
<td colspan="7">AND</td>
</tr>
<tr>
<td colspan="7">limit search to (english language and yr="1979 -Current")</td>
</tr>
</table>

For peer review only

Supplement III

Draft Data Collection Items

Source

- Author
- Title
- Year
- Citation

Eligibility

- Reason for exclusion
- Reason for inclusion
- Answers which question (1-5)

Methods

- Study design
- Study Question/Aim

Population Characteristics

- Number
- Setting
- Age
- Sex
- Country
- Co-morbidity
- Socio-demographics/ethnicity/other

Intervention/Exposure

- Specific Intervention/Exposure
- Comparator
- Number of groups

Outcomes

- Outcomes
- Definition of outcomes/diagnostic criteria used
- Scales/threshold

Results

- Estimate of effect, CIs, p-values

Miscellaneous

- Funding
- Key conclusions
- Comments
- Relevant References

For peer review only
